# Electronic cigarettes: a survey of perceived patient use and attitudes among members of the British thoracic oncology group

**DOI:** 10.1186/s12931-016-0367-y

**Published:** 2016-05-17

**Authors:** Frances C. Sherratt, Lisa Newson, John K. Field

**Affiliations:** Department of Psychological Sciences, University of Liverpool, Whelan Building, Brownlow Hill, Liverpool, , L69 3GB UK; Research Centre for Brain and Behaviour, Natural Sciences and Psychology, Liverpool John Moores University, Tom Reilly Building, Byrom Street, Liverpool, L3 3AF UK; Roy Castle Lung Cancer Research Programme, Department of Molecular and Clinical Cancer Medicine, University of Liverpool, The Apex Building, 6 West Derby Street, Liverpool, L7 8TX UK

**Keywords:** Smoking, Cessation, Electronic cigarettes, e-cigarettes, Lung cancer, Oncologists

## Abstract

**Background:**

Smoking cessation following lung cancer diagnosis has been found to improve several patient outcomes. Electronic cigarette (e-cigarette) use is now prevalent within Great Britain, however, use and practice among patients with lung cancer has not as yet been explored. The current study aims to explore e-cigarette use among patients and examine current practice among clinicians. The results have important implications for future policy and practice.

**Methods:**

Members of The British Thoracic Oncology Group (BTOG) were contacted via several e-circulations (*N =* 2,009), requesting them to complete an online survey. Of these, 7.7 % (*N =* 154) completed the survey, which explored participant demographics and smoking history, perceptions of patient e-cigarette use, practitioner knowledge regarding sources of guidance pertaining to e-cigarettes, and practitioner advice.

**Results:**

Practitioners frequently observed e-cigarette use among patients with lung cancer. The majority of practitioners (81.4 %) reported responding to patient queries pertaining to e-cigarettes within the past year; however, far fewer (21.0 %) felt confident providing patients with e-cigarette advice. Practitioner confidence was found to differentiate by gender (*p* = 0.012) and employment speciality (*p* = 0.030), with nurses reporting particularly low levels of confidence in advising. The results also demonstrate extensive variability regarding the practitioner advice content.

**Conclusions:**

The results demonstrate that patients refer to practitioners as a source of e-cigarette guidance, yet few practitioners feel confident advising. The absence of evidence-based guidance may have contributed towards the exhibited inconsistencies in practitioner advice. The findings highlight that training should be delivered to equip practitioners with the knowledge and confidence to advise patients effectively; this could subsequently improve smoking cessation rates and patient outcomes.

## Background

In 2012, it was estimated there were 1.8 million new lung cancer cases and 1.6 million lung cancer deaths worldwide [1]. Smoking cessation has been identified as one of the most effective strategies to reduce lung cancer incidence [2]. Furthermore, smoking cessation has been found to be highly beneficial among those diagnosed with lung cancer, as continued tobacco smoking following diagnosis has been associated with risk of all-cause mortality, cancer recurrence, and development of a secondary tumour [3].

A recent review highlighted the efficacy of combining pharmacotherapy, such as varenicline, with cognitive and behavioural interventions for smoking cessation among smokers with lung cancer [4]. In the UK, the National Institute for Health and Care Excellence (NICE) produce guidance regarding the diagnosis and treatment of lung cancer, in which they recommend practitioners inform patients of the harms of continuing to smoke tobacco and urge practitioners to advise patients to stop smoking as soon as possible, whilst offering pharmacotherapy [5]. Despite this, 39 % of lung cancer patients are classified as current smokers at diagnosis and 37 % of these patients continue to smoke five months following diagnosis [6], highlighting the importance of exploring attitudes and perceptions of smoking cessation.

To date, NICE fails to provide guidance regarding the use of electronic cigarettes (e-cigarettes) among patients with lung cancer [5] and there is a lack of research in this context. One recent study conducted in the USA, reported e-cigarette use among smokers with lung cancer to be 24 % within the prior 30 days at the point of assessment [7], yet e-cigarette use among patients with lung cancer in Great Britain is currently unclear. The present study partially aims to ascertain observed patient use of e-cigarettes. This is important to inform future policies, research and training offered to practitioners.

A recent review of evidence commissioned by Public Health England suggests that e-cigarettes are approximately 95 % less harmful than regular cigarettes and recommends encouraging smokers struggling to quit, to try e-cigarettes [8], whilst some researchers have expressed concerns regarding uptake of e-cigarettes among people who don’t smoke and about their long term health effect [9]. Furthermore, from May 2016, it will be necessary for e-cigarettes to be licenced within the UK by the Medicines and Healthcare products Regulatory Agency (MHRA) [10], with the aim of improving product safety and efficacy. In relation to e-cigarette use among lung cancer patients, The International Association for the Study of Lung Cancer (IASLC) issued a recent statement [11], in which they recommend that e-cigarette use among lung cancer patients is discouraged, due to the paucity of research pertaining to e-cigarette safety and efficacy.

In light of the absence of evidence-based guidance pertaining to e-cigarette use among patients with lung cancer, this study aimed to explore whether practitioners are aware of the IASLC guidance and in addition, this study examined the prevalence of work-based practitioner guidance on the use of e-cigarettes. This is important to determine, in order to ensure that practitioners are following current guidance and keeping abreast of any research and policy developments, as well as enabling the identification of training needs. Furthermore, the current study aimed to explore the content of the advice that practitioners were providing to patients and to establish whether practitioners were maintaining an evidence-based approach to smoking cessation.

The current study addresses the following questions:

1. How many cancer patients have asked questions about e-cigarettes, have tried them, or report currently using them?
2. Are practitioners aware of sources of e-cigarette guidance (e.g., IASLC guidance)?
3. Are health services in which practitioners are based issuing practitioner guidance regarding e-cigarette use among cancer patients?
4. What advice are practitioners providing to cancer patients regarding e-cigarettes?
5. Do practitioners identify a need for further guidance and support with regards to providing patients with e-cigarette advice and which groups may benefit from further training?

## Methods

### Participants

The British Thoracic Oncology Group (BTOG) represents all disciplines involved in the care of lung cancer and mesothelioma throughout the UK and includes medical and clinical oncologists, respiratory physicians, surgeons, radiotherapists, radiologists, nurses, pharmacists and scientists [12]. Two thousand and nine members of BTOG were contacted, of which 7.7 % (*N* = 154) participants completed the online survey. Of these respondents, seven were excluded as they failed to complete the survey in full; the finalised study sample consisted of 147 BTOG members.

Table 1 demonstrates that the majority of respondents were female (*n* = 95, 64.6 %), between the age range of 30-49 years (*n* = 89, 60.5 %), never smokers (*n* = 103, 70.1 %), and never e-cigarette users (*n* = 132, 90.4 %). Furthermore, nursing was the most frequently cited line of employment (*n* = 52, 35.4 %).

**Table 1:** Sample characteristics and attitudes towards e-cigarettes among BTOG members overall

Variable		Participants n (%)
Age		
	18-29	5 (3.4)
	30-49	89 (60.5)
	50-69	50 (34.0)
	70 or above	3 (2.0)
Gender		
	Female	95 (64.6)
	Male	52 (35.4)
Employment field		
	Clinical oncology	26 (17.7)
	Clinical trials	4 (2.7)
	Lecturer/educator	1 (0.7)
	Medical oncology	11 (7.5)
	Nursing	52 (35.4)
	Palliative care	1 (0.7)
	Pathology	1 (0.7)
	Respiratory	31 (21.1)
	Research/science	4 (2.7)
	Surgery	10 (6.8)
	Other	6 (4.1)
Smoking status		
	Never	103 (70.1)
	Ever	44 (29.9)
E-cigarette use^a^		
	Never	132 (90.4)
	Ever	14 (9.6)
Perceived e-cigarette harm^a^		
	More harmful than regular cigarettes	3 (2.1)
	Equally harmful to regular cigarettes	14 (9.6)
	Less harmful than regular cigarettes	100 (68.5)
	Don’t know	29 (19.9)

^a^Figures do not equate to 147 due to some missing data

### Procedure

Four BTOG e-circulations were posted to 2,009 BTOG members throughout April and May 2015, in which the online survey was detailed and members were requested to participate. The online survey was open between April 1^st^ 2015 and May 31^st^ 2015. Members were provided with a web-link, which directed them to a participant information sheet, consent form, the online questionnaire, and subsequently, a debrief form, in which participants were directed to a link to the IASLC statement regarding e-cigarette use among cancer patients [11].

### Measures

All participants were asked several questions pertaining to socio-demographics, smoking history and e-cigarette use and risk perception. Gender and age were firstly ascertained. Employment speciality was assessed based on the categories presented on the membership page of the BTOG website: [13] “Please describe your speciality” (Clinical oncology, Clinical trials, Lecturer/educator, Medical oncology, Nursing, Pharmacy, Palliative care, Pathology, Radiology, Respiratory, Research/science, Surgery, Other). Smoking status was ascertained using measures from the Behavioral Risk Factor Surveillance System: [14] These measures included: “Have you smoked at least 100 cigarettes in your entire life?” (Yes, No) and “Do you now smoke cigarettes every day, some days, or not at all?” (Every day, Some days, Not at all). The variable was subsequently collapsed to categorise ever and never smokers. E-cigarette ever-use was also ascertained, using a measure adopted in previous studies: [15–17] “Have you ever tried an electronic cigarette?” (Yes, No). E-cigarette risk perception was assessed by adapting and utilising a measure applied in previous research: [18–20] “Do you think that electronic cigarettes are more harmful than regular cigarettes, less harmful, or are they equally harmful to health?” (More harmful than regular cigarettes, Equally harmful to regular cigarettes, Less harmful than regular cigarettes, Don’t know). Lastly, patient contact was established, in order to assess participant suitability for additional patient-related measures: “In your profession, do you have contact with patients?” (Yes, I have patient contact, No, I do not have patient contact).

Participants who reported patient contact were asked a further three questions pertaining to observed e-cigarette use among patients, which were adapted from similar measures of observed e-cigarette use among Stop Smoking Service users: [21, 22] (1) “What proportion of patients who are current and former smokers you have seen in the past year have asked you questions about electronic cigarettes (e-cigarettes)?”; (2) “What proportion of patients who are current and former smokers you have seen in the past year say they have ever used e-cigarettes?”; (3) “What proportion of patients who are current and former smokers you have seen in the past year say they regularly use e-cigarettes?”. The response options to each of these three questions, included “None”, “Less than a quarter”, “From a quarter to a half”, “From a half to three quarters”, and “More than three quarters”.

Participants were asked five further questions, regarding sources of e-cigarette guidance, content of advice provided to patients, and practitioner confidence in advising patients regarding e-cigarettes. The majority of these questions were informed by previous research: [21, 22] Does your workplace have a recommendation of what advice you should give patients on electronic cigarettes? (Yes, No); What advice do you give patients about e-cigarettes? (Open text box of 500 characters); To what extent do you agree with the following statement: “I feel I need more information and guidance regarding electronic cigarettes” (Strongly agree, Agree, Neutral, Disagree, Strongly disagree). The remaining two questions were developed by the research group, which consisted of professionals with clinical and research expertise in the fields of lung cancer and e-cigarettes. These final questions included: Are you aware of the statement The International Association for the Study of Lung Cancer (IASLC) released regarding electronic cigarettes and cancer patients? (Yes, No), and; To what extent do you agree with the following statement: “I feel confident advising patients regarding electronic cigarettes” (Strongly agree, Agree, Neutral, Disagree, Strongly disagree). The aforementioned variable levels for the measure which explored practitioner confidence advising patients regarding e-cigarettes were refined prior to bivariate analyses, due to low cell frequencies; “Strongly agree” and “Agree” were combined to form “Agree”, “Neutral” remained the same, and “Strongly Disagree” and “Disagree” were combined to form “Disagree”.

### Ethical approval

The University of Liverpool provided full ethical approval for the study (Reference: RETH000832); all participants provided informed consent, were made aware that they could withdraw from the study at any time, data was anonymised, strict confidentiality guidelines were adhered to, and participants were aware that the results derived from the data may be published in a scientific journal. Furthermore, upon submission of the online questionnaire, participants were directed to a link to the IASLC statement regarding e-cigarette use among cancer patients [11].

### Data analysis

As described, the study primarily aimed to explore perceived prevalence of e-cigarette use among patients, whilst considering practitioners’ knowledge regarding sources of guidance pertaining to e-cigarettes, confidence in advising patients, and the content of patient advice provided. Univariate analyses were primarily undertaken to achieve these aims. The questionnaire included several open-ended questions as described, which offered participants the opportunity to enter free text. These brief free text responses were entered into a spreadsheet and coded; responses were coded initially by the primary coder (FS) and the results were corroborated by a second coder (LN). Further bivariate analyses were additionally undertaken to explore differences between practitioner confidence in advising patients across socio-demographic and smoking-related variables, using *χ*^2^ test or Fisher’s exact test as appropriate for categorical variables. All statistical analyses were performed using IBM SPSS Statistics for Windows Version 21.0 (Armonk, NY).

## Results

Most participants perceived e-cigarettes to be safer than regular cigarettes (see Table 1) (*n* = 100, 68.5 %). Participants who reported patient contact (*n* = 141, 96.6 %) were subsequently asked several questions pertaining to patient e-cigarette use; the associated responses are detailed in Table 2. A large proportion of practitioners had been asked about e-cigarettes by patients who were ever-smokers within the past year; only 18.7 % (*n* = 25) of practitioners reported no patients having asked about e-cigarettes within the past year, whilst, a small number reported more than three quarters of patients having asked about e-cigarettes within the past year (*n* = 4, 3.0 %). E-cigarette ever-use also appeared to be prevalent in consideration of practitioner responses, as almost half reported ever-use of e-cigarettes among 25 % of more of their ever-smoking patients (*n* = 62, 42.4 %), whilst a small number reported none of their patients ever having used e-cigarettes (*n* = 6, 4.6 %). Similarly, regular e-cigarette use appeared prevalent among patients; approximately a third of practitioners estimated regular use of e-cigarettes among 25 % or more of the ever-smoking patients they had seen in the past year (*n* = 42, 32.6 %).

**Table 2:** Practitioners’ responses to questions regarding e-cigarette use among patients with thoracic malignancies

Variable			Participants *n* (%)
Proportion of ever-smoker patients who have:			
	Asked questions regarding e-cigarettes^a^		
		None	25 (18.7)
		Less than a quarter	69 (51.5)
		From a quarter to half	25 (18.7)
		From a half to three quarters	11 (8.2)
		More than three quarters	4 (3.0)
	Ever-used e-cigarettes^a^		
		None	6 (4.6)
		Less than a quarter	63 (48.1)
		From a quarter to half	47 (35.9)
		From a half to three quarters	12 (9.2)
		More than three quarters	3 (2.3)
	Regularly use e-cigarettes^a^		
		None	8 (6.2)
		Less than a quarter	79 (61.2)
		From a quarter to half	38 (29.5)
		From a half to three quarters	3 (2.3)
		More than three quarters	1 (0.8)

^a^Figures do not equate to 141 due to some missing data

BTOG members with patient contact (*n* = 141) were asked further questions regarding sources of e-cigarette guidance and advice they provided to patients (see Table 3). The results demonstrated that the majority of participants were not aware of the IASLC statement pertaining to e-cigarette use among cancer patients (*n* = 97, 72.4 %) and furthermore, most reported a lack of workplace recommendations regarding e-cigarette use among cancer patients (*n* = 122, 91.0 %). Participants additionally reported poor levels of confidence regarding providing patients with e-cigarette-related advice. The vast majority of participants agreed or strongly agreed that they needed more information and guidance regarding e-cigarettes to advise patients (*n* = 124, 92.6 %), whilst only a small proportion of participants felt confident advising patients regarding e-cigarettes (*n* = 28, 21.0 %).

**Table 3:** Practitioners’ responses to questions regarding sources of e-cigarette guidance and reported advice provided

Variable		Participants *n* (%)
Awareness of IASLC statement regarding e-cigarette use among cancer patients^a^		
	Yes	37 (27.6)
	No	97 (72.4)
Workplace recommended advice that practitioners should provide regarding e-cigarettes^a^		
	Yes	12 (9.0)
	No	122 (91.0)
“I feel I need more information and guidance regarding electronic cigarettes”^a^		
	Strongly agree	53 (39.6)
	Agree	71 (53.0)
	Neutral	4 (3.0)
	Disagree	6 (4.5)
	Strongly disagree	0 (0.0)
“I feel confident advising patients regarding electronic cigarettes” ^a^		
	Strongly agree	4 (3.0)
	Agree	24 (18.0)
	Neutral	39 (29.3)
	Disagree	49 (36.8)
	Strongly disagree	17 (12.8)
Advice given to patients regarding e-cigarettes^b^		
	E-cigarettes are less harmful than regular cigarettes	45 (23.7)
	Paucity of research and uncertainty regarding adverse effects	41 (21.6)
	Patients should avoid using regular or electronic cigarettes altogether	25 (13.2)
	E-cigarettes may be an effective tool for smoking cessation	20 (10.5)
	E-cigarette use is discouraged	11 (5.8)
	Seek support via Stop Smoking Services	10 (5.3)
	Lack of regulation and caution regarding quality control	10 (5.3)
	No advice provided	9 (4.7)
	E-cigarette use is encouraged	7 (3.7)
	Consider the use of licenced smoking cessation treatments primarily	5 (2.6)
	E-cigarettes may be harmful to health	3 (1.6)
	Inadequate knowledge to advise	3 (1.6)
	No clear guidelines from professional bodies	1 (0.5)

^a^Figures do not equate to 141 due to some missing data, ^b^More than one answer could be provided

The results also demonstrated inconsistencies regarding the content of the advice provided to patients by practitioners (Table 3). Most frequently, practitioners advised that e-cigarettes were likely to be less harmful that regular cigarettes (*n* = 45, 23.7 %) and that there is a paucity of research and uncertainty regarding adverse effects associated with e-cigarette use (*n* = 41, 21.6 %). Some practitioners provided no advice or suggested that they had inadequate knowledge to advise patients (*n* = 12, 6.3 %). Furthermore, some explicitly described how they would encourage (*n* = 7, 3.7 %) or conversely, discourage patient e-cigarette use (*n* = 11, 5.8 %).

Bivariate associations between practitioner confidence in advising patients regarding e-cigarettes and several participant characteristics were examined. Practitioner confidence in advising patients was found to differentiate significantly by employment speciality (*p* = 0.030). Nurses were most likely to disagree that they felt confident advising patients (*n* = 33, 50.0 %), whilst those who reported their employment speciality as respiratory were most likely to agree that they felt confident advising patients (*n* = 10, 35.7 %). Male practitioners were more likely to exhibit confidence in advising patients, compared to females (*p* = 0.012) (see Table 4). Practitioner confidence in advising patients was not found to be significantly related to age, smoking status or e-cigarette status.

**Table 4:** Associations between practitioner confidence advising patients regarding electronic cigarettes and demographic and smoking-related variables

Variable		Confident advising patients regarding e-cigarettes			
		Agree *n* (%)	Neutral *n* (%)	Disagree *n* (%)	*p*-value
Age^a^					0.651
	18-29 years	0 (0)	1 (2.6)	2 (3.0)	
	30-49 years	16 (57.1)	26 (66.7)	41 (62.1)	
	50-69 years	12 (42.9)	10 (25.6)	41 (62.1)	
	70 years and above	0 (0)	2 (5.1)	1 (1.5)	
Gender^a^					0.012*
	Male	17 (60.7)	13 (33.3)	19 (28.8)	
	Female	11 (39.3)	26 (66.7)	47 (71.2)	
Employment speciality^a^					0.030*
	Nursing	5 (17.9)	12 (30.8)	33 (50.0)	
	Respiratory	10 (35.7)	9 (23.1)	12 (18.2)	
	Clinical Oncology	9 (32.1)	7 (17.9)	7 (10.6)	
	Medical Oncology	1 (3.6)	5 (12.8)	5 (7.6)	
	Surgery	3 (10.7)	2 (5.1)	4 (6.1)	
	Other	0 (0)	4 (10.3)	5 (7.6)	
Smoking status^a^					0.969
	Never	8 (28.6)	11 (28.2)	20 (30.3)	
	Ever	20 (71.4)	28 (71.8)	46 (69.7)	
E-cigarette status^a^					0.633
	Never	3 (10.7)	3 (7.7)	4 (6.1)	
	Ever	25 (89.3)	36 (92.3)	62 (93.9)	

^a^Figures do not equate to 141 due to some missing data, * *p* < 0.05

## Discussion

This is the first study, to our knowledge, to: (1) estimate e-cigarette prevalence among patients with lung cancer in Great Britain, and; (2) explore practice associated with e-cigarette use among cancer patients. These components are important to investigate, as the associated findings contribute towards the development of future research and guidance, as well as informing future training delivered to practitioners working with cancer patients who smoke.

There were a number of key findings in the current study. The current study highlighted that the vast majority of practitioners are being asked by their patients (those diagnosed with lung cancer) about e-cigarettes. Furthermore, the majority of practitioners frequently observed e-cigarette ever-use among patients. Use of e-cigarettes in the USA among patients with lung cancer was recently estimated to be 24 %; [7] the current results appear to reflect these statistics, as observed patient use of e-cigarettes was most often estimated to be below 25 %. The findings not only ascertain frequent e-cigarette use among patients but they demonstrate how a substantial proportion of patients view practitioners as a source of advice and guidance regarding e-cigarettes and smoking cessation.

Despite prevalent use and frequent patient queries pertaining to e-cigarettes, the survey revealed typically poor levels of practitioner confidence regarding advising patients on e-cigarettes. The results demonstrated how practitioner confidence also differentiated by employment speciality and gender, suggesting that specific groups could benefit from further training and support in advising patients. Poor confidence was also reflected by the lack of awareness of the aforementioned IASLC statement and limited workplace recommendations pertaining to e-cigarette use among cancer patients.

Extensive variability was additionally evident across practitioner advice, with several conflicting examples of practitioner advice being identified. Furthermore, practitioners appeared substantially more optimistic regarding e-cigarette harm compared with the general public; 69 % of practitioners in the current study perceived e-cigarettes to be less harmful than regular cigarettes, whilst only 52 % of the British general population were recently estimated to perceive e-cigarettes as less harm than regular cigarettes [23].

Prior research suggests that cancer patients perceive practitioner advice regarding quitting as a key reason for smoking cessation [24], whilst practitioner support has been associated with smoking cessation success [25]. Practitioners have, however, been found to exhibit personal beliefs regarding practice, which can conflict with evidence-based guidance and ultimately impact practice [26–28]. The current results suggest that due to the absence of evidence-based e-cigarette guidance or potentially, lack of training, practitioners could be projecting their personal beliefs regarding e-cigarette use onto patients. Inconsistent practice is of great concern, as this could ultimately affect patient outcomes. Although research continues to aim to ascertain the safety and efficacy of e-cigarettes, the reported low levels of practitioner confidence identified in the current study, might be improved by increasing e-cigarette-related training among practitioners and increasing awareness of sources of e-cigarette guidance for both practitioners and cancer patients.

The current study has a number of limitations. Firstly, objective data were not available regarding patient e-cigarette use. Although the current study provides an estimate of e-cigarette use among patients with lung cancer, some patients may not have accurately conveyed e-cigarette use to practitioners, which could have resulted in an underestimation of patient use.

Secondly, there was a limited response rate from BTOG members compared to some other surveys of thoracic oncology providers [29] and therefore, data analysis options were limited and the sample may not have been fully representative of BTOG membership. This may have been due to a lack of perceived e-cigarette importance or clinical time constraints. It should be noted that the study design entailed a self-selected sample and findings could therefore result in potential bias, however, this is a limitation of any self-selecting online survey [30]. A larger survey or qualitative study may now be helpful to verify these findings and to explore strategies designed to improve practitioners’ knowledge and confidence regarding advising patients about e-cigarettes.

This study highlights the extreme disparities between practitioners in relation to the guidance provided and also suggests that current guidance is either not being accessed or is being ignored. The disparity that exists among practitioners warrants further consideration. The MHRA intends to regulate e-cigarettes in May 2016 [10], which should improve attempts to ascertain product safety and efficacy. Whilst further research explores the efficacy and safety of e-cigarettes in this context, efforts should be made to ensure regular training and support for practitioners regarding e-cigarette use among cancer patients, particularly across employment specialities that demonstrate low confidence in advising patients (e.g., nursing); in doing so, practitioner competence and confidence advising patients regarding e-cigarettes should increase, which could improve smoking cessation rates and in turn, patient outcomes. Furthermore, guidance regarding e-cigarette use among cancer patients should be updated regularly, in line with the rapidly evolving e-cigarettes evidence-base; this will promote the provision of up-to-date and accurate e-cigarette practitioner advice.

## Conclusion

This is the first study, to our knowledge, that attempts to estimate e-cigarette prevalence among cancer patients in Great Britain and explores practice associated with e-cigarette use among cancer patients. The study revealed the prevalence of both e-cigarette use and associated patient-practitioner queries. Although the results highlighted that patients rely upon practitioners as a source of guidance regarding e-cigarettes, practitioners typically demonstrated low levels of confidence in guiding patients and poor awareness of sources of guidance pertaining to e-cigarette use among cancer patients. The findings have important implications for practitioner training, as well as future research and policy. Effective and consistent practitioner advice regarding e-cigarettes and smoking cessation could improve smoking cessation rates and subsequently, improve patient outcomes.

## Abbreviations

E-cigarette: Electronic cigarette
BTOG: British Thoracic Oncology Group
IASLC: International Association for the Study of Lung Cancer
MHRA: Medicines and Healthcare products Regulatory Agency
